# P300 event-related potentials as diagnostic biomarkers for attention deficit hyperactivity disorder in children

**DOI:** 10.3389/fpsyt.2025.1590850

**Published:** 2025-08-05

**Authors:** Chuanxue Tan, Huimin Zhou, Anqi Zheng, Miao Yang, Chunyang Li, Ting Yang, Tingyu Li, Jie Zhang

**Affiliations:** ^1^ Department of Child Health Care, Xi’an Children’s Hospital, Xi’an, China; ^2^ Children’s Nutrition Research Center, Children’s Hospital of Chongqing Medical University, Chongqing Key Laboratory of Child Neurodevelopment and Cognitive Disorders, Ministry of Education Key Laboratory of Child Development and Disorders, National Clinical Research Center for Child Health and Disorders, Chongqing, China; ^3^ Department of Radiology, Xi’an Children’s Hospital, Xi’an, China

**Keywords:** ADHD, event-related potentials, P300, ROC Curve, diagnostic biomarkers

## Abstract

**Objectives:**

This study aimed to evaluate the utility of P300 event-related potentials (ERPs) as neurophysiological biomarkers for diagnosing attention deficit hyperactivity disorder (ADHD) and to establish preliminary diagnostic thresholds for their use.

**Methods:**

A total of 106 children diagnosed with ADHD and 66 healthy controls were enrolled. Using a visual Oddball paradigm, P300 parameters were recorded at Fz, Cz, and Pz zones. Key metrics analyzed included P300 amplitude and latency as well as reaction time and correct responses. Statistical tests and logistic regression analysis identified significant group differences, while receiver operating characteristic (ROC) analysis determined the diagnostic performance of these parameters.

**Results:**

Children with ADHD exhibited significantly lower P300 amplitudes and longer latencies across all electrode sites compared to controls. Logistic regression identified Cz amplitude (p = 0.001), Pz amplitude (p = 0.011), maximum reaction time (p = 0.037), and correct response count (p < 0.001) as significant predictors of ADHD. ROC analysis showed that Cz amplitude, Pz amplitude, maximum reaction time, and correct responses achieved AUCs of 0.81, 0.75, 0.72, and 0.86, respectively, with sensitivities ranging from 66% to 80% and specificities from 61% to 95%. These results underscore the diagnostic potential of both electrophysiological and behavioral markers in ADHD assessment.

**Conclusions:**

Cz and Pz amplitude, maximum reaction time, and correct responses each demonstrated strong diagnostic utility for distinguishing ADHD from typically developing children. The use of these neurophysiological and behavioral indicators as objective complements to traditional clinical assessments.

## 1 Introduction

Attention deficit hyperactivity disorder (ADHD) is one of the most prevalent neurodevelopmental disorders, affecting approximately 7% of children worldwide (1). ADHD is associated with long-term consequences, including higher rates of academic failure, occupational challenges, and increased risk of psychiatric comorbidities (2, 3). Despite its high prevalence, the diagnosis of ADHD primarily relies on subjective behavioral assessments, such as the DSM-5 criteria and parental or teacher rating scales (4). While these methods are valuable, their reliance on subjective interpretation introduces variability and limits their accuracy (5).

In recent years, there has been growing interest in identifying objective neurophysiological biomarkers to complement existing diagnostic methods. Neurophysiological measures, particularly event-related potentials (ERPs), have emerged as promising tools (6). ERPs are time-locked brain responses to specific stimuli that provide insights into cognitive processes such as attention, memory, and executive functioning (7). Among these, the P300 component has garnered significant attention due to its sensitivity to attentional and cognitive control mechanisms. P300 is typically elicited using an Oddball paradigm, where participants respond to infrequent target stimuli amid frequent non-target stimuli. The P300 waveform is characterized by a positive deflection occurring approximately 300 milliseconds post-stimulus, with its amplitude and latency reflecting cognitive engagement and information processing speed, respectively (8).

Research has consistently shown that children with ADHD exhibit abnormal P300 parameters, including reduced amplitude and prolonged latency (9). Studies indicate that these ERP abnormalities may stem from dysfunctions in the prefrontal cortex and parietal regions, which are critical for cognitive control and attention regulation (10, 11). Despite these promising findings, the use of P300 in clinical practice remains limited. This is largely due to challenges such as the lack of normative reference data, variability in EEG acquisition and analysis protocols across laboratories, technical and resource-related constraints, inconsistent thresholds for interpretation and limited understanding of P300’s diagnostic utility (12, 13).

Against this background, this study investigates whether P300 ERP parameters can serve as objective markers for diagnosing ADHD in children. By integrating ERP features with behavioral performance measures (reaction time and accuracy), we move beyond group comparisons to assess their diagnostic utility. P300 amplitude and latency were measured at midline electrodes (Fz, Cz, Pz) using a visual oddball paradigm. This study is among the first to apply ROC analysis to combine ERP and behavioral data, aiming to derive preliminary diagnostic thresholds with improved classification accuracy. We hypothesize that children with ADHD will show significantly lower P300 amplitudes and longer latencies than controls, and that combining ERP and behavioral metrics will enhance diagnostic precision.

## 2 Materials and methods

### 2.1 Study population

This study included 172 children aged 6–12 years who were recruited from outpatient services at the Child Health Department of the local children’s hospital. Pediatric neurologists diagnosed all participants based on the DSM-5 criteria through structured clinical interviews conducted and divided into an ADHD group (n=106) and a control group (n=66). Inclusion criteria required participants to have an IQ ≥85 (assessed using WISC-V) and no history of neurological or psychiatric disorders (14, 15). The control group comprised typically developing children with no history of neurodevelopmental, psychiatric, or neurological disorders. Exclusion criteria included 1) sensory impairments, such as uncorrected vision or hearing loss;2) comorbid neurological disorders;3) history of traumatic brain injury;4) prior use of stimulant medications. Ethical approval for the study was obtained from the local hospital. Written informed consent was obtained from parents or guardians, and assent was provided by children when appropriate.

### 2.2 Experimental procedures

**Figure 1:**
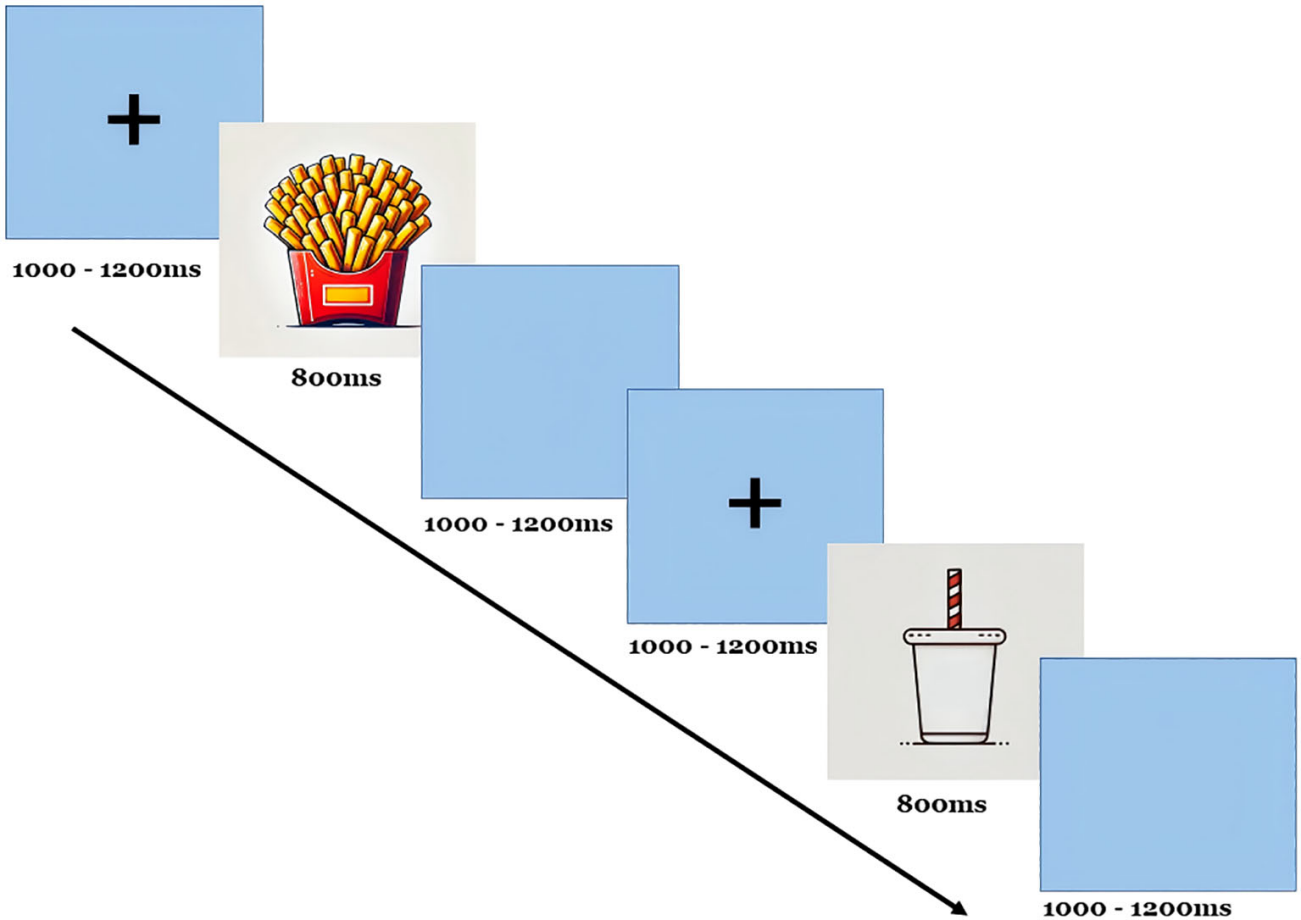
Oddball procedures for ERP.

Our experiment followed a typical visual oddball paradigm, as depicted in **Figure 1**, and was executed using E-prime software for both compilation and control (16). All subjects completed the experiment using only one hand (right-handed). In this task, the “French fries” pattern served as the target stimulus, while other patterns acted as non-target stimuli. Each stimulus was displayed for 800 ms, with intervals of 1000–1200 ms between presentations. A total of 300 stimuli were shown randomly, with target stimuli comprising 20% and non-target stimuli making up 80%. Participants were instructed to respond by pressing a key when a target stimulus appeared and refraining from pressing when a non-target stimulus was shown. EEG data were segmented into 1000 ms epochs, spanning from –150 ms before stimulus onset to 850 ms after. A baseline correction was applied using a fixed pre-stimulus window from –150 ms to –100 ms.

Participants were instructed to sit upright and maintain open eyes throughout the task. They were asked to count the target stimuli and respond accordingly. The task was performed in a soundproof, dimly lit room to reduce distractions, ensuring a controlled environment for recording the EEG. The EEG was recorded using a 32-channel electrode cap with placements following the International 10–20 system. Key electrode sites for P300 analysis included Fz (frontal), Cz (central), and Pz (parietal). Additional electrodes were placed to monitor vertical eye movements for artifact correction. The study recorded several indicators, including latency, amplitude, maximum reaction time, minimum reaction time, average reaction time, and the number of correct responses (hits) for target stimuli.

EEG preprocessing and artifact correction were performed using EEGLAB running on MATLAB (MathWorks Inc.). Continuous EEG data were bandpass filtered between 0.1 and 30 Hz and then segmented into epochs time-locked to stimulus onset. Ocular artifacts, including blinks and saccades, were removed using Independent Component Analysis (ICA) via EEGLAB’s runica algorithm. Components corresponding to eye movements were identified based on their scalp topographies and time-course features and were manually removed. All data were visually inspected after ICA to confirm artifact removal (17).

### 2.3 Quality control

To ensure the validity and reliability of the study, several quality control measures were implemented. All participants with ADHD were diagnosed by two senior pediatricians from the Children’s Health Center at Xi’an Children’s Hospital, following DSM-V criteria through interviews. The principal investigator provided detailed instructions to parents before filling out the questionnaires to guarantee the authenticity and reliability of the results. During the P300 recording process, participant information (name, age, gender, and outpatient number) was documented.

To prevent bias in data processing, all EEG datasets were anonymized and coded by participant ID before analysis. The researchers involved in preprocessing (artifact rejection, ICA, ERP measurement) were blinded to participants’ diagnostic group (ADHD vs. control) throughout the analysis. To ensure the validity and reliability of the data, if significant artifacts were detected during the P300 recording (such as eye movements or muscle interference), only the problematic trials were discarded. No participant underwent the task more than once, and the number of stimulus presentations remained constant across all participants. The total task duration was approximately 7–8 minutes, with participants completing a standard set of 300 stimuli (60 target stimuli and 240 non-target stimuli).

Any participant showing excessive habituation effects, such as reduced response accuracy or diminished P300 amplitude across the task, was excluded from the final analysis. Only those with stable performance throughout the task were retained. Trials were excluded if they showed excessive noise, defined by a peak-to-peak amplitude exceeding ±100 μV, non-biological artifacts (e.g., high-frequency bursts), or loss of signal (flatlines). Visual inspection was also conducted by trained analysts to confirm ERP waveform integrity and remove residual contaminated epochs. After data collection, the data were audited and entered into the EpiData dual-entry database system, and logical verification was performed to identify and correct any inconsistencies or errors.

### 2.4 Statistical analysis

All data in this study were analyzed using SPSS 27.0 statistical software. Demographic, clinical and EEG variables were assessed for normality using the Shapiro-Wilk test and the presence of outliers prior to statistical analysis. Descriptive analysis was conducted for each study variable, with numerical variables expressed as mean, standard deviation, minimum, and maximum values. Group differences in P300 amplitude and latency were analyzed using t-tests for normally distributed data. Non-normally distributed data were analyzed using Mann-Whitney U tests or Kruskal-Wallis tests. To evaluate the diagnostic utility of P300 parameters, univariate and multivariate logistic regression analyses were conducted. Odds ratios (ORs) with 95% confidence intervals (CIs) were reported for each variable. ROC analysis evaluated the diagnostic performance of P300 parameters, with sensitivity, specificity, and area under the curve (AUC) calculated for different thresholds. Optimal cutoffs were determined using the Youden index. A p-value of less than 0.05 was considered statistically significant.

## 3 Results

### 3.1 Demographic characteristics

The demographic and baseline characteristics of the study participants are presented in **Table 1**. The ADHD group included 60% male participants, with a mean age of 9.3 ± 2.1 years, and a mean IQ of 103.8 ± 11.2. Similarly, the control group consisted of 58% males, with a mean age of 9.2 ± 1.9 years and a mean IQ of 105.4 ± 10.5. No significant differences were observed in age, gender distribution, or full-scale IQ scores between the ADHD and control groups (p > 0.05).

**Table 1:** The demographic characteristics of the study population.

Characteristic	ADHD Group (n=106)	Control Group (n=66)	p-value	Cohen’d/ OR
Age (years)	9.3 ± 2.1	9.2 ± 1.9	0.78	-0.55
Male	62.26%	68.18%	0.85	1.65
IQ (Full-scale)	103.8 ± 11.2	105.4 ± 10.5	0.34	0.15

ADHD, attention deficit hyperactivity disorder.

### 3.2 P300 difference between ADHD and healthy controls

**Figure 2:**
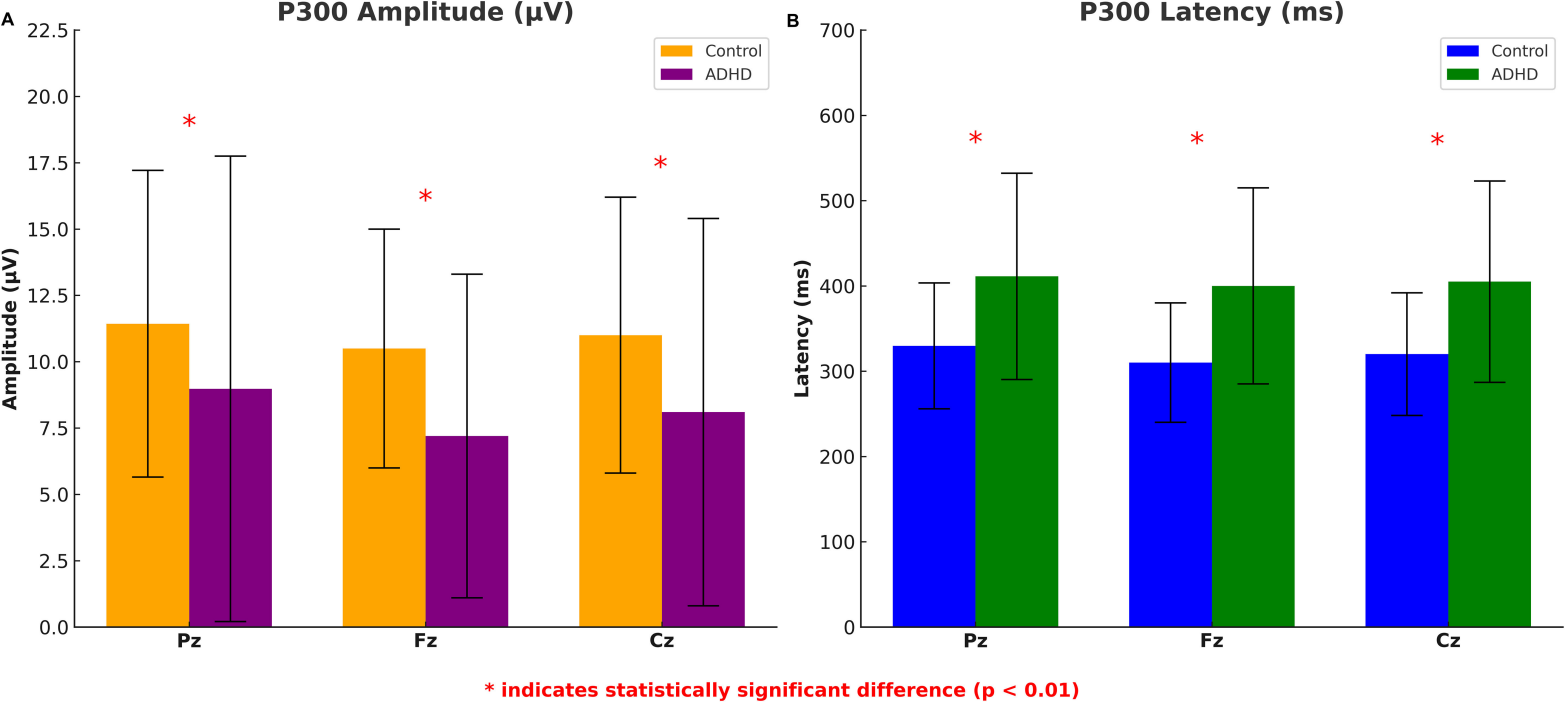
P300 amplitude and latency differences between ADHD group and healthy controls. The error bars represent standard deviations.

Children with ADHD exhibited significantly lower P300 amplitudes across all zones compared to the control group. At the Fz, Cz and Pz zones, the mean amplitude for the ADHD group (4.59 ± 3.30 μV, 4.07 ± 3.02 μV, 4.13 ± 3.10 μV) were significant lower compared to the control group (7.88 ± 3.38 μV, 7.87 ± 3.89 μV, 9.45 ± 5.86 μV, p = 0.002; p = 0.001; p = 0.003, respectively) (**Figure 2A**). P300 latency was significantly prolonged in the ADHD group com-pared to controls. At the Pz zone, the mean latency for the ADHD group was 411.19 ± 122.12 milliseconds, whereas the control group demonstrated a latency of 330.65 ± 74.47 milliseconds (p = 0.003). Similar patterns of prolonged latency were observed at the Fz and Cz zones compared to controls (p = 0.004; p = 0.004) (**Figure 2B**).

**Figure 3:**
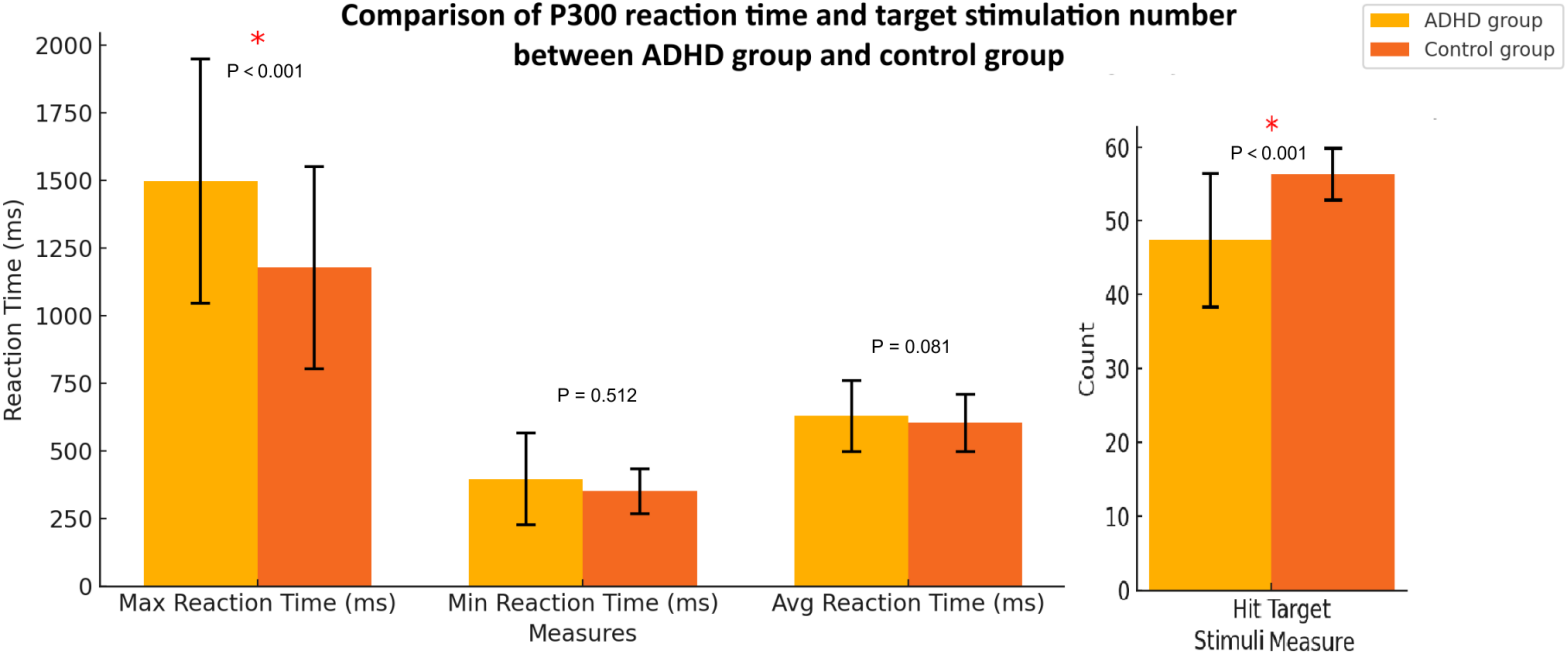
P300 response times and number of correct responses (hits) for target stimuli between the ADHD group and health controls. The error bars represent standard deviations.

The comparison of P300 response times and number of correct responses (hits) for target stimuli between the ADHD group and the control group showed that the ADHD group had significantly higher maximum reaction time (1497.21 ± 453.88 ms) and fewer correct responses (47.40 ± 9.08) than that in the control group (1179.09 ± 378.54 ms; 56.29 ± 3.57, respectively) with statistically significant (p < 0.001, respectively). The minimum reaction time, and average reaction time were also higher than the control group without statistically significant (p = 0.512; p = 0.081, respectively) (**Figure 3**).

### 3.3 Logistic regression analyses

**Table 2** presents the univariate and multivariate logistic regression analyses of P300-related parameters between ADHD and non-ADHD groups. In the univariate analysis, significant differences were observed in all eight parameters: ADHD participants exhibited prolonged latencies at Fz, Cz, and Pz zones (all p < 0.001) and reduced amplitudes at Fz, Cz, and Pz zones (all p < 0.001, except Pz amplitude p = 0.744). The ADHD participants had slower maximum response times (p < 0.001) and fewer target stimulus hits (p < 0.001). Multivariate logistic regression identified Cz amplitude (p = 0.001), maximum response time (p = 0.037), number of correct responses (p < 0.001), and Pz amplitude (p = 0.001) as significant predictors of ADHD.

**Table 2:** Univariate and multivariate regression for ADHD and non-ADHD.

P300 features	Outcome			Univariate regression		Multivariate regression	
	ADHD(Means ± SD)	Non-ADHD (Means ± SD)	Cohen’s d	Unadjusted OR (95% CI)	P-Value	Adjusted OR (95% CI)	P-Value
Fz Latency	399.03 (114.96)	335.32 (73.82)	0.659	1.008 (1.004–1.011)	<0.001	1.003 (0.995-1.012)	0.444
Cz Latency	398.74 (117.28)	332.95 (87.89)	0.635	1.006 (1.003–1.010)	<0.001	1.000 (0.993–1.006)	0.911
Pz Latency	411.40 (122.12)	330.65 (74.47)	0.798	1.009 (1.005-1.014)	<0.001	1.007 (1.000–1.015)	0.053
Fz Amplitude	4.59 (3.30)	7.88 (3.38)	-0.985	0.744 (0.667-0.831)	<0.001	1.017 (0.841-1.229)	0.865
Cz Amplitude	4.07 (3.02)	7.87 (3.89)	-1.091	0.683 (0.601–0.776)	<0.001	0.702 (0.569–0.865)	*0.001*
Pz Amplitude	4.13 (3.10)	9.45 (5.86)	-1.135	0.675 (0.598–0.762)	<0.001	0.720 (0.598–0.868)	*0.001*
Max Response Time (ms)	1497.21 (453.88)	1179.09 (378.54)	0.761	1.002 (1.001-1.003)	<0.001	1.001 (1.000–1.002)	*0.037*
Target Stimulus Hits	47.40 (9.08)	56.29 (3.57)	-1.289	0.719 (0.643-0.804)	<0.001	0.766 (0.672–0.872)	*<0.001*

### 3.4 ROC curve analysis

**Figure 4:**
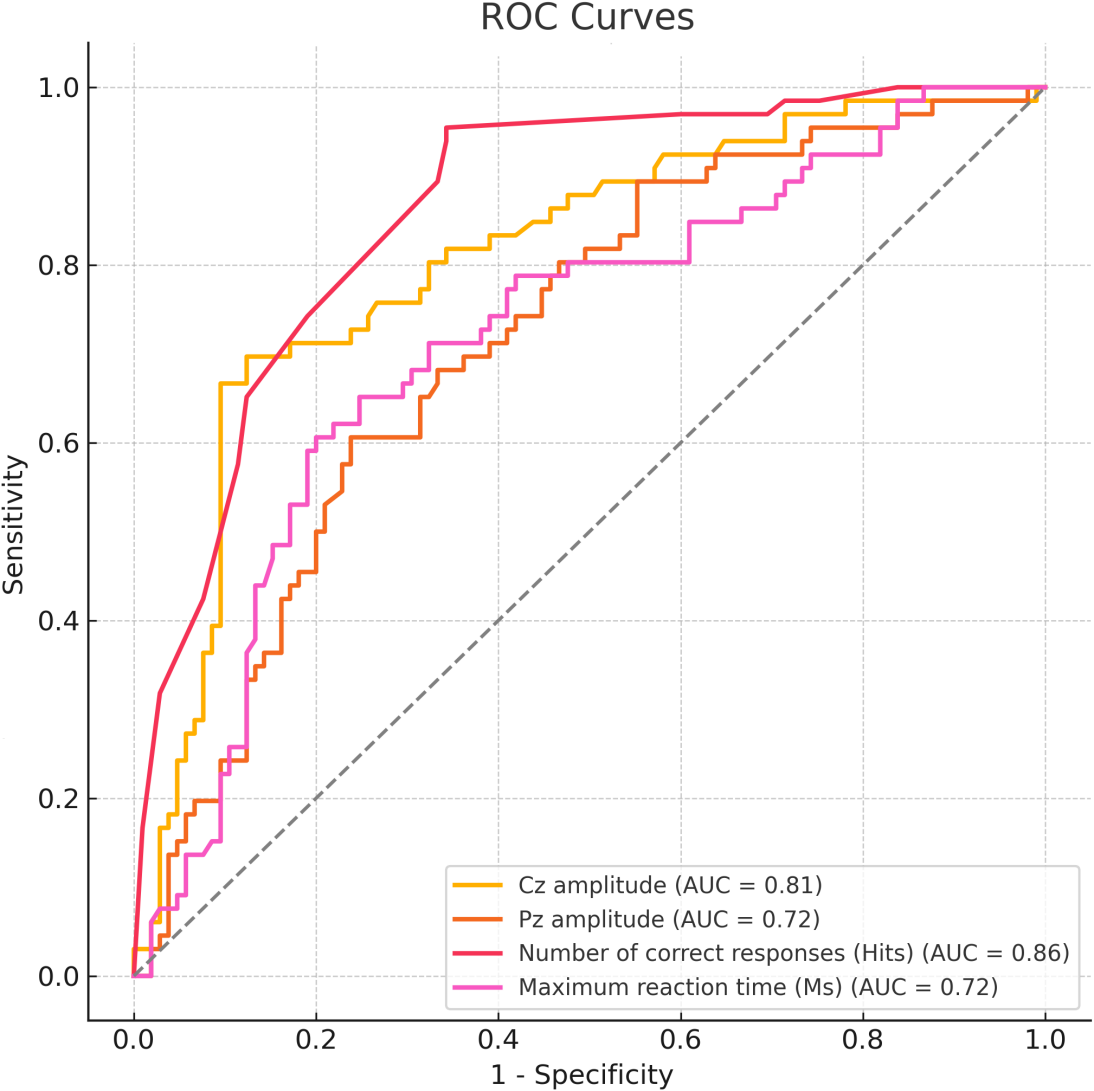
ROC curve analysis for P300 parameters between ADHD group and health controls.

ROC curve analysis was performed to evaluate the diagnostic value of Cz amplitude, Pz amplitude, number of correct responses (hits), and maximum reaction time (RT) in distinguishing ADHD from controls. The number of correct responses showed the highest accuracy (AUC = 0.86; cut-off = 51; sensitivity = 95.5%; specificity = 65.7%), followed by Cz amplitude (AUC = 0.81; cut-off = 7.21 μV; sensitivity = 69.7%; specificity = 87.6%). Pz amplitude and RT, after directional correction, yielded moderate performance (both AUCs = 0.72), with cut-offs of 42.80 μV and 1231 ms, respectively (**Figure 4**).

## 4 Discussion

In recent decades, the diagnosis of ADHD in children and adolescents has risen significantly, making it one of the most common pediatric psychiatric disorders. The het-erogeneity in clinical presentation presents challenges for accurate diagnosis (18). This variability includes frequent comorbidities, symptom overlap with other neurodevelopmental and psychiatric disorders, and context-dependent symptoms. As a result, behavioral questionnaires, such as those based on DSM-5 criteria, often lead to inconsistent diagnostic accuracy and variable correlation with disease severity. These inconsistencies, widely reported in the literature, emphasize the need for objective and reliable biomarkers (19, 20). This study utilized the P300 component of ERP, known for its sensitivity to ADHD-related cognitive processes, to effectively differentiate children with ADHD from healthy controls using the oddball paradigm.

This study identified eight significant P300 parameters distinguishing ADHD from healthy controls, including P300 latency and amplitude across Cz, Fz, and Pz regions, maximum reaction time, and correct response count. These measures reflect core neurophysiological and cognitive deficits associated with ADHD. The prolonged P300 latency in the ADHD group likely indicates delayed neural processing in brain regions essential for attention regulation and cognitive control, such as the prefrontal cortex and parietal lobes. These delays may be due to structural and functional abnormalities commonly seen in ADHD, such as reduced gray matter volume and disrupted attentional network connectivity. Additionally, the reduced P300 amplitude in ADHD participants suggests impaired allocation of attentional resources and lower cognitive engagement during tasks. This supports the hypothesis that ADHD is linked to deficits in the neural mechanisms responsible for salience attribution and executive function, as evidenced by studies connecting P300 abnormalities to prefrontal dysfunction (21, 22). It is well established that individuals with ADHD often exhibit higher intra-individual variability in reaction times (IVRT) compared to healthy controls, even in the absence of significant differences in mean reaction time (23). In our study, the maximum reaction time was significantly higher in the ADHD group, which may reflect the underlying increased IVRT. This suggests that the observed reaction time variability could be a critical measure of attentional and cognitive control deficits in ADHD (24). Future studies should consider incorporating IVRT as an additional diagnostic measure to better understand ADHD-related variability in cognitive processing.

The logistic regression analysis in this study provided key insights into the predictive value of P300 parameters for diagnosing ADHD. In the univariate analysis, all eight parameters showed significant differences between the ADHD and control groups. The multivariate logistic regression, which considered potential interactions among multiple predictors, revealed that Cz amplitude, Pz amplitude, maximum reaction time, and the number of correct responses were significant factors for ADHD diagnosis. The significance of both Cz and Pz amplitudes suggests that these midline electrodes capture complementary aspects of ADHD-related neural dysfunction. Cz amplitude likely reflects impairments in central attentional control and motor integration, whereas Pz amplitude may be more sensitive to deficits in posterior attention allocation and stimulus evaluation. This co-significance aligns with neurocognitive models of ADHD that emphasize dysfunction across distributed fronto-parietal attention networks (4, 5). Hence, both components are crucial for accurately characterizing the disorder**’**s neurophysiological profile.

Behavioral deficits, such as prolonged reaction times and fewer correct responses, highlight the challenges ADHD children face in sustaining attention and responding to tasks. Delayed reaction times are likely due to disrupted information processing in brain regions like the prefrontal cortex and basal ganglia, affecting attentional control and motor response initiation (21, 21). Impaired connectivity between these areas and motor regions, such as the supplementary motor area, further exacerbates these delays (25). Additionally, the reduced number of correct responses indicates difficulties in sustained attention and impulse control, especially when inhibiting competing stimuli (22). This difficulty in detecting target stimuli reflects impairments in salience attribution and executive control, involving the anterior cingulate cortex and parietal lobes (20). Delayed P300 responses also contribute to these deficits by limiting the child**’**s ability to allocate attentional resources during tasks.

The strong discriminative power of correct responses and Cz amplitude likely reflects their close association with core cognitive deficits in ADHD. Behavioral accuracy captures sustained attention and executive control, both of which are commonly impaired in ADHD. Reduced correct responses reflect lapses in focus and task engagement. Meanwhile, the Cz electrode records activity from fronto-central regions involved in cognitive control, such as the anterior cingulate cortex. A lower P300 amplitude at Cz indicates impaired attentional allocation and context updating, consistent with prior findings. Cz may thus provide an optimal neural marker by balancing sensitivity to executive function with lower signal variability, contributing to its superior classification accuracy (26).

Despite the strengths of this study, several limitations should be noted. First, the small sample size (children aged 6–12 years from a single center) limits the generalizability of the findings. Larger, multi-center validation studies are needed to confirm the robustness of the biomarkers. Future studies should include a broader demographic adjustment, particularly for sex, which was not accounted for in the current model due to data limitations. Additionally, the cross-sectional design does not assess the biomarkers**’** predictive value over time, highlighting the need for longitudinal studies to evaluate their ability to predict treatment response or developmental trajectories. Our ongoing research aims to expand the sample size and include follow-ups to enhance understanding. The absence of correlation analysis between the biomarkers and standardized neuropsychological scales is another limitation. Future research should explore these relationships to improve clinical relevance and applicability. Diagnostic thresholds derived from ROC analysis were not validated through cross-validation or bootstrapping, and should therefore be considered preliminary and sample-specific. In this study, we focused on analyzing the P300 peak amplitude as it is widely used in ADHD research. However, analyzing the mean amplitude within a defined time window (e.g., 250–400 ms) may provide a more stable measure, particularly by reducing variability due to noise or artifacts. Future studies could explore both approaches to assess their relative effectiveness in distinguishing ADHD from healthy controls, and to enhance diagnostic accuracy. Additionally, scalp topography maps and mean ERP waveforms with standard error indicators can provide important insights into group-level patterns of neural activation. Future work will incorporate these visualizations to enhance the spatial resolution and interpretability of ERP data in ADHD. Finally, while our study excluded children with psychiatric comorbidities and controlled for major neurological confounders, we did not assess participants**’** psychosocial stress levels prior medication history, subthreshold comorbidities or exposure to adverse life events. Given the growing evidence linking stress to altered attention and ERP responses, future studies should incorporate standardized assessments of psychosocial factors to more accurately isolate ADHD-specific biomarkers.

In conclusion, this study highlights the diagnostic relevance of individual P300-related parameters in ADHD. Cz and Pz amplitudes, maximum reaction time, and correct response count each demonstrated meaningful discriminative value. These neurophysiological and behavioral measures may serve as objective, quantifiable complements to traditional behavioral assessments in the clinical evaluation of ADHD.

## Data Availability

The datasets generated and analyzed during the current study are available from the corresponding author on reasonable request.
